# Performance of Graphite-Dispersed Li_2_CO_3_-K_2_CO_3_ Molten Salt Nanofluid for a Direct Absorption Solar Collector System

**DOI:** 10.3390/molecules25020375

**Published:** 2020-01-16

**Authors:** M. A. Karim, Majedul Islam, Owen Arthur, Prasad KDV Yarlagadda

**Affiliations:** Science and Engineering Faculty, Queensland University of Technology, Brisbane, QLD 4001, Australia; azharul.karim@qut.edu.au (M.A.K.); o.arthur@live.com (O.A.); y.prasad@qut.edu.au (P.K.Y.)

**Keywords:** molten salt nanofluids, direct absorption solar collector, computational fluid dynamics

## Abstract

Considered to be the next generation of heat transfer fluids (HTFs), nanofluids have been receiving a growing interest over the past decade. Molten salt nanofluids have been shown to have great potential as an HTF for use in high temperature applications such as direct absorption solar collector (DAC) system. Very few studies using molten salt nanofluids as the HTF in a DAC receiver can be found in the open literature. This study aimed to develop a 3D computational fluid dynamics model of the receiver of a DAC using graphite-nanoparticle-dispersed Li_2_CO_3_-K_2_CO_3_ molten salt nanofluid to investigate the effects of design and operation parameters on receiver performance. Receiver total efficiency using Li_2_CO_3_-K_2_CO_3_ salt was compared with that using solar salt nanofluid. Spectral properties of the base fluid and nanoparticles were modeled as wavelength-dependent and the absorption of the solar radiation was modeled as a volumetric heat release in the flowing heat transfer fluid. Initial results show that the receiver efficiency increases with increasing solar concentration, decreasing nanoparticle volume fraction, and decreasing receiver length. It was also found that the Carnot efficiency increases with increasing receiver length and nanoparticle volume fraction, and decreasing solar concentration and inlet velocity. Comparative study shows that solar salt HTF could provide higher total efficiency. However, a higher operating temperature of Li_2_CO_3_-K_2_CO_3_ will allow for a greater amount of thermal energy storage for a smaller volume of liquid.

## 1. Introduction

Heat transfer fluids (HTFs) are critical to concentrating solar power (CSP) plants, and their selection is paramount to the overall efficiency of the system [1,2,3,4,5,6,7]. Overall energy efficiency of the CSP electricity generation plants are limited by the operating temperature of the HTF as per the general thermodynamic cycle. The Carnot efficiency jumps from 50 to 65% if the operating temperature is increased from 300–400 °C to 560 °C [8]. This low operating temperature is one of the main obstacles for the CSP technology to compete with conventional fossil fuel technology that works above 1300 °C [9]. To reach an unsubsidized parity with fossil fuels, a heat capacity and operating temperature of 2.25 kJ/kgK and 600–800 °C, respectively, are needed for an HTF fluid in place of the current state of the art 1.5kJ/kgK and 228–565 °C [10]. Water, glycol, synthetic oil, and molten salts are common HTFs. Water and glycol, while having good thermal conductivity and specific heat, cannot be used in high temperature CSP applications because of their low boiling points, 100 and 177 °C, respectively, at 1 atm pressure [11]. The boiling point of the commonly used synthetic oil, Therminol VP-1, is also not that high, only 390 °C [12]. Moreover, the oil has undesirable properties of high vapor pressure and breaks down into hydrogen upon decomposition. On the contrary, molten salts can reach temperatures as high as 600 °C. Moreover, they are naturally abundant, cheap, and mostly safe for the environment. However, they suffer from poor thermophysical properties; for instance, the specific heat is generally less than 2 kJ/kgK [12].

Nanofluids-suspension of nano-sized solid particles in a liquid, since its coining in 1995 by Choi, has been emerging as a new alternative HTF [13]. Unlike micron-sized suspensions, nanofluids form stable colloidal suspensions with next to no settling under static conditions [14]. Increase in the thermal conductivity and, in some instances, increase in the specific heat capacity were observed for these suspensions compared to that of the base fluid [15]. With the use of nanofluids, the receiver efficiency and the Carnot efficiency can be improved.

Concentrated solar radiation can be focused onto a black or spectrally selective coated high-absorptive receiver of a conventional solar thermal collector, which can then be conducted to an HTF to be used in a thermodynamic cycle [16]. These conventional receivers suffer from major drawbacks at high temperatures including (i) a lower conversion efficiency because of a non-uniform and high temperature gradient between the receiver wall and the HTF, (ii) significant convective and radiative losses from the bare receiver to the environment, lowering the overall energy efficiency, and (iii) significant thermal stress on the material, causing it to degrade the absorber [17]. 

Instead, a direct absorption collector (DAC), conceptually somewhat similar to small particle collectors, can directly absorb solar radiation into the HTF, resulting in a more uniform heat distribution and low temperature gradient between the absorber and fluid [18,19,20,21]. Being in the order of nanometer, the small particles can be fluidized to pass through pumps, micro-channels, and piping in the form of nanofluid without any adverse effects, and even a low volume concentration could significantly improve the solar radiation absorption of the DAC [22]. 

Using a nanofluid receiver in place of a conventional surface type receiver, an efficiency improvement in the order of 5–10% is possible [22]. With the recent advancements in nanotechnology, nanofluid DACs have gained significant interest because of the following advantages:

- The nanoparticles dominate the optical properties of the nanofluid, which allows us to tune the performance of the receiver by altering the size, shape, concentration, and material type of the particles [22].
- As the nanofluid directly absorbs the solar radiation, DACs do not require a surface absorption plate [23].
- Nanofluids possess superior thermophysical properties, including an enhanced thermal conductivity, heat transfer coefficient, and, in some cases, specific heat capacity [8,24,25,26].
- Optically selective nanofluids allow for high absorption in the solar range and low emittance in the infrared. Therefore, a volumetric receiver can be employed in place of a selective surface receiver of higher emissive losses resulting from a poorer temperature profile [27].

While direct sunlight is incident on a thin flowing film of water/aluminum nanofluid, a 2D finite difference heat transfer model assuming Rayleigh scattering, developed by Tyagi et al., shows that approximately 10% efficiency can be increased using a DAC receiver over a conventional flat-plate solar collector [28]. However, their computational model was not verified experimentally. Otanicar et al. validated the model experimentally and studied the size-dependent effects on the nanoparticle optical properties [29]. They observed a steep initial increase in receiver efficiency of up to 5% before it leveled off as the volume fraction continued to increase. The discrepancy between this result with the previous result was attributed to the agglomeration and sedimentation of the nanofluid, and to the fact that more concentrated light will be absorbed in a thin upper layer of the nanofluids that can be easily transferred back out of the receiver. Luo et al. also found a 2–25% increased collector efficiency using nanofluids in comparison to the base fluid for their experimentally validated simulation model [30]. Parvin et al. also found a 31% enhancement in heat transfer performance using nanofluid, and the DAC efficiency was more than doubled [31]. From a 2D CFD study, Kaluri et al. observed an increase in efficiency of up to 28% from a DAC considering the effects of optical concentration, the optical density of fluid, the mass flow rate, and the thermal insulation on the receiver efficiency [17]. 

While enhancement in the optical properties of an HTF by nanoparticles is evident [32,33], its thermophysical properties including the thermal conductivity and, in some cases, the specific heat can be affected, too. As the specific heat of water-based nanofluids has been found to decrease with the addition of nanoparticles, most investigations of this have focused on the enhancement of thermal conductivity of water-based and glycol-based nanofluids for cooling applications [34,35]. However, recent studies confirmed anomalous enhancements in specific heat capacity of some non-aqueous nanofluids. For instance, Tiznobaik and Shin, Shin and Banerjee, Shin and Tiznobaik, and Shin et al. reported a maximum increase in specific heat capacity of 29, 24, 124, 22.37, and 26%, respectively, compared to the base fluid for lithium carbonate–potassium carbonate doped with ${\mathrm{SiO}}_{2}$ nanoparticles [8,15,36,37,38]. Recently, with the same base fluid, lithium carbonate–potassium carbonate, Shin and Banerjee reported a 31–33% enhancement in the heat capacity with aluminum oxide (Al_2_O_3_) nanoparticle doping [39]. Yang and Banerjee, and Shin and Banerjee, observed a maximum increase in specific heat capacity of 13 and 14.5%, respectively, compared to the base fluid alkali metal chloride salt eutectics doped with ${\mathrm{SiO}}_{2}$ nanoparticles [40,41]. Similarly, after doping the ${\mathrm{SiO}}_{2}$ nanoparticles in sodium nitrate–potassium nitrate salts (NaNO_3_–KNO_3_), Budda and Shin, Chieruzzi et al., and Jung and Banerjee reported an increase in the specific heat capacity by 28, 57.7, and 18.6%, respectively [42,43,44].

Another potential HTF is a nanoparticle-enhanced ionic fluid that consists of organic-based compounds with discrete charges resulting in a significantly lower vapor pressure. Being a type of molten salt, several ionic fluids can be found to have low freezing points (below 0 °C), are non-volatile at atmospheric pressure, possess relatively high heat capacities, and are approachable to high temperature (about 400 °C) [45]. Many researchers have investigated the thermophysical properties of ionic fluids experimentally. Dispersing Al_2_O_3_ in ionic liquids, [C_4_ mim][BETI], [C_4_ mim][NTf_2_], [C_4_ mmim][NTf_2_], and [C_4_ mpyrr][NTf_2_], it was found that the specific heat and the thermal conductivity were increased by 9–32% and by 3–6%, respectively. However, the disperse had an adverse effect on the HTFs’ viscosity, which was reported to increase by 20% on average and, in some cases, up to 70% [12,24,45,46,47]. Similar investigations were accomplished using dispersing graphene nanoparticles in a range of nanofluids with multi-walled carbon nanotubes. The nanofluids include these previously mentioned ones plus [C_4_ mim][BF_4_], [C_4_ mim][CF_3_SO_3_], [C_6_ mim][NTf_2_], [C_8_ mim][NTf_2_] and [HMIM]BF_4_. In these cases, though the thermal conductivity was found to be increased (ranging between 4 and 35.5%) as expected, the specific heat capacity was observed to be decreased slightly (ranging between 0.2 and 2.4%). However, the viscosity of [HMIM]BF_4_ nanofluids was observed to be decreased unexpectedly with the addition of graphene nanoparticles and multi-walled carbon nanotubes compared to the base fluid. The recorded decrease was up to 19% in some cases [48,49,50].

The upper temperature limit and the thermophysical properties of nanofluids are two of the most important factors to consider for concentrating solar thermal collector systems. The upper temperature limit is important as it determines the maximum Carnot efficiency of the total system and directly influences the efficiency of any thermal storage that might be included in the system. The thermophysical properties are important as they affect how the heat flows, directly influencing the efficiency of the solar receiver and the thermal storage.

Apart from ionic fluid, molten salt nanofluids have also been investigated experimentally as base fluids dispersing different nanoparticles for high temperature applications. The molten salts mostly used have been sodium nitrate–potassium nitrate salts (NaNO_3_–KNO_3_, commonly known as solar salt) and lithium carbonate–potassium carbonate (Li_2_CO_3_–K_2_CO_3_). The nanoparticles used with the solar-salt-based fluids were SiO_2_ [42,43], Al_2_O_3_, TiO_2_, SiO_2_–Al_2_O_3_ [43], and Mica [44], and those with lithium-salt-based fluids were SiO_2_ [36,37,38,51], Al_2_O_3_ [39], graphite [52,53], and multi-walled carbon nanotubes [38,54,55,56]. Most of these research works investigated the effect of thermophysical properties, including the thermal conductivity, specific heat, and viscosity. 

However, no to little studies using graphite-dispersed molten salt nanofluids are available in the open literature. In addition to this, the application of this nanofluid as the HTF in a DAC receiver was introduced for the first time in this study. Using a verified 3D CFD model of a DAC receiver, developed using COMSOL Multiphysics 5.3 (COMSOL Inc., Burlington, MA 01803, USA) engineering software package, we investigated the energy performance of graphite-doped solar salts nanofluids for high temperature applications, as published earlier [6]. This study was further extended to investigate the performance of graphite suspended Li_2_CO_3_–K_2_CO_3_ molten salt nanofluid as an HTF for a DAC receiver. The model was validated by comparing the results to others found in the literature. Factors of the receiver to be taken into account are the thermal re-radiation of the HTF to the environment, convective and conductive heat transfer with the environment, forced convection due to wind, the volume fraction of nanoparticles, the size of the nanoparticles, and the receiver geometry. The parametric study includes the effect of the receiver length, the inlet velocity, the volume fraction, and the concentration of the incident solar radiation on the Carnot, receiver, and total efficiencies of the receiver. The comparative effect of Li_2_CO_3_–K_2_CO_3_ and solar salts on the total efficiency of the receiver were also investigated.

## 2. Computational Model Development and Solution Procedure

### 2.1. Model Setup and Boundary Conditions

**Figure 1:**
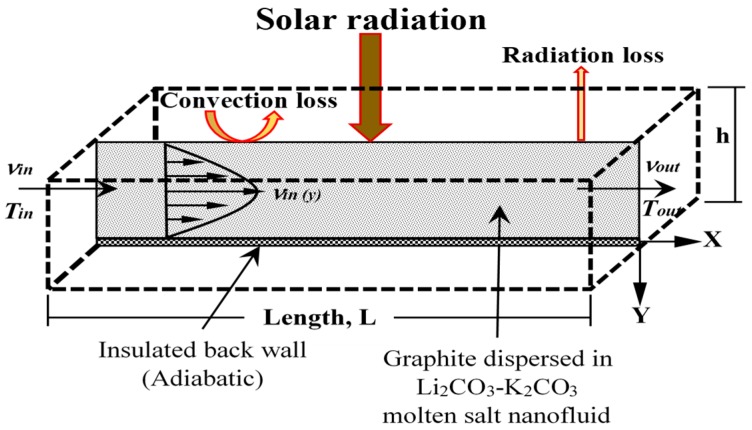
Computational model setup for the direct absorption collector (DAC) receiver.

The model setup for the DAC receiver is shown in Figure 1. The model shows that graphite nanoparticle-suspended Li_2_CO_3_–K_2_CO_3_ molten salt nanofluid as an HTF is flowing between two parallel, horizontal, and no-slip flat plates of height h. It is assumed that the concentrated sun is incident normally and uniformly on the HTF through the top transparent wall of the DAC receiver, the bottom wall is perfectly insulated (i.e., adiabatic), the receiver is volumetrically heated, the HTF flow is fully developed, and the flow and energy variation across the width of the receiver is negligible. The side walls of the receiver were modeled as planes of symmetry. Therefore, though the model is 3D to account for a volumetric heat source, it is 2D in nature. Heat losses are included, namely, convection and radiation from the top surface to the ambient, as shown in the figure, and thermal re-emission from within the fluid. The bottom of the receiver is insulated and modeled as an adiabatic black wall, and reflective losses are not considered.

The adiabatic surface, symmetry, and outflow boundary conditions were simulated as

(1) −n⋅(−k∇T)=0.

The opaque surface is simulated as

(2) $$-n\cdot \left(-k\nabla T\right)=q_{r,net}$$

(3) $$I_{i,bnd}=\varepsilon_{W}{/}_{b}\left(T\right)+\frac{1-\varepsilon_{W}}{\pi }q_{r,net},n\cdot S_{i}<0$$

(4) $$q_{r,out}=\sum_{n\cdot S_{j}>0}\omega_{j}{/}_{j}n\cdot S_{j}.$$

The temperature boundary condition was used to simulate the inlet temperature of the receiver.

(5) $$T=T_{0}$$

Heat flux is calculated using

(6) $$-n\cdot \left(-k\nabla T\right)=h\cdot \left(T_{ext}-T\right).$$

### 2.2. Thermo-Physical and Rheological Properties of the Nanofluid

This study considers a mixture of Li_2_CO_3_–K_2_CO_3_ at a mole fraction of 62:38, respectively. Graphite, a commonly occurring dopant in the literature, is used as a nanoparticle with this molten salt to produce the HTF. Graphite nanoparticles exhibit high absorptivity in the solar range. The operating temperature range of the HTF is an important factor for a power generation cycle for maximizing its Carnot efficiency. Graphite-doped Li_2_CO_3_–K_2_CO_3_ nanofluid is very well suited to high-temperature DAC systems. Li_2_CO_3_–K_2_CO_3_ has a melting point of 761 K and an upper temperature limit of 1071 K; above this temperature, the salt becomes unstable [57]. Therefore, care has been taken to ensure that the operating temperature limit of the HTF lies between 780 and 1071 K. The temperature-dependent thermophysical properties, except the *C_p_*, of the HTF for this temperature limit are employed as shown by Equations (7) to (9) [57]. *C_p_* is set to 1600 J/kgK. Because of a very low volume concentration, the influence of the nanoparticles on the thermophysical properties of the nanofluid is neglected for simplicity. The nanofluid is considered to be a Newtonian fluid in nature [58,59,60]:

(7) $$\rho_{nf}=1991-0.4341T\;\left(\frac{\mathrm{kg}}{m^{3}}\right)$$

(8) $$k_{nf}=0.618+0.000948T\;\left(\frac{W}{m.K}\right)$$

(9) $$\mu_{nf}=\frac{e^{-1.0473+\frac{1781}{T}}}{1000}\;\left(\mathrm{Pa}.s\right)$$

where T is in Kelvin.

The Krieger–Dougherty model is used to calculate the viscosity of the nanofluid as shown in Equation (10).

(10) $$\mu_{nf}=\mu_{bf}{\left(1-\frac{\phi_{a}}{\phi_{m}}\right)}^{1\left[\eta \right]\phi_{m}}$$

### 2.3. Modeling Optical Properties of the Nanofluid

Particle shape, size, material type, and volume fraction of the dopant have a significant influence on the optical properties of the nanofluid. As no reliable theory exists to describe the impact of varying particle shapes, except for the spherical ones on the optical properties of the nanofluid [35], it is assumed that the nanoparticles are spherical in shape. A size parameter, *α*, is employed to quantify the size effect of the nanoparticle as per Equation (11).

(11) $$\alpha =\frac{\pi \;D}{\lambda }.$$

A combination of refractive and absorptive indexes is used to quantify the complex refractive index of different material [34].

(12) $$s_{np}=n_{np}+ia_{np}.$$

The Mie theory can be used to account for the absorption and scattering of spheres, [36,37]. However, Rayleigh type scattering can be assumed for the particles with a diameter smaller than the wavelength of light in a medium. The extinction efficiency is the combination of the absorption efficiency and the scattering efficiency of the HTF.

(13) $$Q_{ext}=Q_{abs}+Q_{scat}=\left[4\alpha Im\;\left(\frac{m^{2}-1}{m^{2}+2}\right)\left\{1-\frac{4\alpha^{3}}{3}Im{\left(\frac{m^{2}-1}{m^{2}+2}\right)}^{2}\right\}\right]+\left[\frac{8\alpha^{4}}{3}{\left|\frac{m^{2}-1}{m^{2}+2}\right|}^{2}\right].\;$$

Due to an extremely small size ratio, |m|α≪1, many of the higher order components in Mie scattering theory can be ignored [31,32]. Again, dependent scattering effects can be ignored for volume fractions less than 0.6% [33]. Therefore, for nanoparticles, the extinction efficiency can be defined by Equation (14).

(14) $$Q_{ext}=Q_{abs}=4\alpha Im\;\left(\frac{m^{2}-1}{m^{2}+2}\right).$$

As can be seen in Equation (11), this simplification is only valid for sufficiently uniform small particles for the fraction of incident light that is scattered [24,36].

(15) $$\frac{I_{s}}{I_{0}}\approx \frac{\pi^{4}ND^{6}}{8\lambda^{4}r^{2}}{\left|\frac{m^{2}-1}{m^{2}+2}\right|}^{2}\left(1+\cos^{2}\vartheta \right).$$

Since the base fluid is not completely transparent, the total extinction coefficient of the nanofluid under the assumption that no aggregation occurs can be represented by Equation (16) [24].

(16) $$\kappa =\kappa_{np}+\kappa_{bf}=\left[\frac{3\emptyset Q_{abs}}{2D}\right]+\left[\frac{4\pi \alpha_{bf}}{\lambda }\right].$$

The concentrated solar radiation can be approximated using Planck’s black body distribution.

(17) $$I_{0}\left(\lambda \right)=S_{att}c\Omega_{s}\frac{2h_{p}c^{2}}{\lambda^{5}}\frac{1}{e^{\frac{hc}{\lambda k_{B}T_{sun}}}-1},\;200\;\mathrm{nm}\leq \lambda \leq 2500\;\mathrm{nm}.$$

### 2.4. Governing Equations 

In order to account for both fluid dynamics and heat transfer into the DAC receiver, two physics modules in COMSOL Multiphysics are incorporated. A fully developed velocity profile at the inlet is assumed considering that the nanoparticles are suspended in the fluid. For the mixture model, the governing equations are from Equation (18) to Equation (27).

(18) $$\rho \left(u\cdot \nabla \right)u=\nabla \cdot \left[-pl+\mu \left(\nabla u+{\left(\nabla u\right)}^{T}\right)\right]-\nabla \cdot \left[\rho c_{d}\left(1-c_{d}\right)u_{slip}u_{slip}\right]+\rho_{c}\left(\nabla \cdot u\right)$$

(19) $$\nabla \cdot N_{\phi_{d}}=-\frac{m_{dc}}{\rho_{d}}$$

(20) $$N_{\phi_{d}}=\phi_{d}u_{d}$$

(21) $$u_{d}=u+\left(1-c_{d}\right)u_{slip}$$

(22) $$\rho =\phi_{c}\rho_{c}+\phi_{d}\rho_{d}$$

(23) $$c_{d}=\frac{\phi_{d}\rho_{d}}{\rho }$$

(24) $$3\frac{C_{d}\rho_{c}}{4d_{d}}\left|u_{slip}\right|u_{slip}=\frac{-\left(\rho -\rho_{d}\right)}{\rho }\nabla p$$

(25) Rep=ddρc|uslip|μ

(26) Cd=24Rep.

The heat transfer can be defined by Equation (27).

(27) $$\rho C_{p}u\cdot \nabla T=\nabla \cdot \left(k\nabla T\right)+Q.$$

As the Reynolds number of a volumetric receiver generally varies between 10 and 1000 [24,39], a fully developed flow has been used in this study. However, an early study modeled the flow within the receiver as a plug flow assuming a creeping flow for a Reynolds number less than 1 or an inviscid flow [16,24,39]. The slip velocity between the nanoparticles and the base fluid is modeled as per the Hadamard–Rybcynski model [61]. Because of the high surface-to-volume ratio, heat transfer between the nanoparticles and the base fluid is assumed as an instantaneous and near-zero temperature gradient between the mediums [41]. The volumetric heat release for a normally incident, negligible scattering and quasi-steady state is given as

(28) $$Q\left(y\right)=-\int_{\lambda_{\min}}^{\lambda_{\max}}\kappa_{\lambda }\left(I_{b\lambda }\left(T\right)-I_{0,\lambda }\right)\;e^{-\kappa_{\lambda }y}dy$$

where *y* is the distance from the top of the receiver, and $I_{0,\lambda }$ is the concentrated normally incident solar radiation quantified using Planck’s black body distribution.

Stefan–Boltzmann’s law is applied to account for the radiative heat loss from the receiver top surface to the ambient.

(29) $$q=\varepsilon \;\sigma \left(T_{amb}^{4}-T^{4}\right).$$

An overall heat transfer coefficient is calculated using Equation (30) in order to calculate convection heat loss.

(30) $$\frac{1}{h_{total}}=\frac{1}{h_{nf}}+\frac{t}{k_{tp}}$$

where *h_nf_* is the convection coefficient between the nanofluid and the top surface, and *k_tp_* is the heat conductivity of the top plate.

The top plate of the receiver is modeled as a fused quartz with a 1 cm thickness for good thermal insulation, as its thermal conductivity is only 1.3 W/mK. Moreover, the material is stable at high temperatures with an annealing point of 1140 °C, has an extremely low coefficient of thermal expansion that reduces the effects of thermal shock, and, most importantly, is virtually transparent in the solar range with an absorptive index ranging from 1.72 × 10^−6^ to 1.354 × 10^−5^ [39].

The average Nusselt number of the nanofluid is given by Equation (31), from which the heat transfer coefficient, *h*, can be calculated.

(31) Nu=0.664Re12Pr13

(32) $$h=\frac{Nu\;k}{L}.$$

Solar radiation that reaches the bottom surface of the receiver, being an adiabatic black wall (as assumed), is completely absorbed and released as heat energy as per Equation (33).

(33) $$P_{bottom,surface}=\int_{\lambda_{\min}}^{\lambda_{\max}}\left(I_{0,\lambda }e^{-\kappa_{\lambda }y_{rec}}+I_{b\lambda }\left(T\right)\left(1-e^{-\kappa_{\lambda }y_{rec}}\right)\right)d\lambda .$$

Finally, the receiver efficiency is calculated as the ratio of usable thermal energy to the incident solar energy.

(34) $$\eta_{rec}=\frac{\dot{m}C_{P}\left(T_{out}-T_{in}\right)}{I_{0}A_{r}}=\frac{\rho_{bf}\nu_{in}y_{rec}w_{rec}C_{P,bf}\left(T_{out}-T_{in}\right)}{P_{y,0}l_{rec}w_{rec}}\;.$$

### 2.5. Solution Procedure

**Figure 2:**
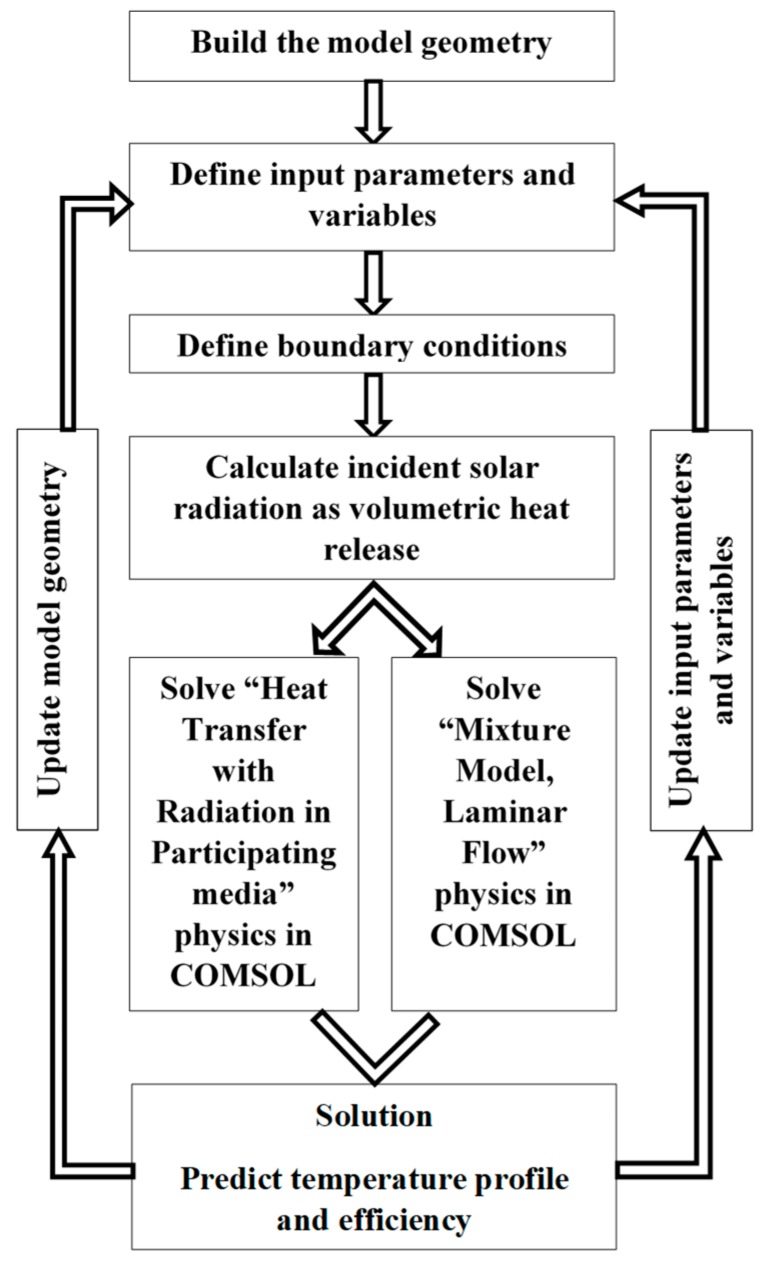
COMSOL Multiphysics modeling strategy.

The solution method of the governing equations using heat transfer with radiation in participating media, and the mixture model, Laminar flow physics in COMSOL, is shown in Figure 2. The incident solar radiation is calculated as the volumetric heat release. The figure shows the solution strategy is a feedback-based interactive process between two different physics including fluid flow physics and radiative energy transfer between participating mediums. Relative tolerance was set to 1 × 10^−5^, and the default absolute tolerance, 1 × 10^−4^, was used for all dependent variables. To make this model converge quickly, the tolerance for the pressure was set to 1 × 10^−3^. However, at the end of the solution, the residual values were found to be much smaller than the set value.

### 2.6. Model Parameters and Variables

The work of Veeraragavan et al. [51] is chosen for the current computational model for validation purposes. Therefore, the parameters used in the literature is used for the current model as presented in Table 1. On the other hand, the variables considered in this simulation are listed in Table 2 with their expressions.

### 2.7. Grid Generation

**Figure 3:**
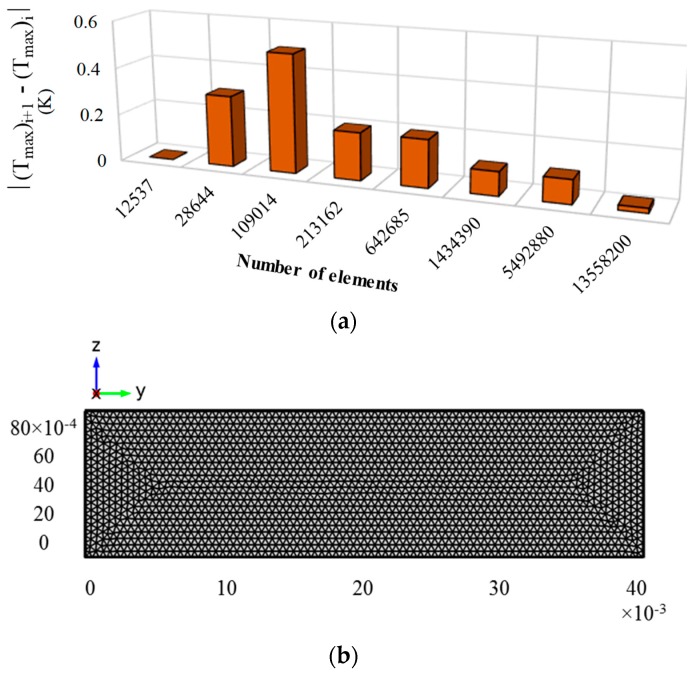
(**a**) Stability of the maximum static temperature of the receiver for different grid systems and (**b**) a generated mesh for the Therminol model.

A grid independence test was performed recording variation in the maximum static temperature (K) from the model for eight different grid systems as shown in Figure 3a. From the test, it was found that a grid system with elements equal to or more than 54,928,84 is quite sufficient to produce a reasonably stable result for the current model. A typical grid system used in this model is presented in Figure 3b. 

### 2.8. Model Validation

Veeraragavan et al. [51] presented an analytical model for the design of volumetric solar flow receivers using graphite-dispersed Therminol nanofluids. Their model was a 2D parallel plate configuration, where the top plate had thermal losses and the bottom plate was perfectly insulated for no heat transfer. Moreover, the absorbed radiation was modeled as a volumetric heat release. Because of the similarity with the current model, the study of Veeraragavan et al. [51] was recreated for validation purpose. To enable the recreated results to be as similar as possible to the boundary conditions, the governing equations and input parameters of the current model are kept the same or similar where possible. 

**Figure 4:**
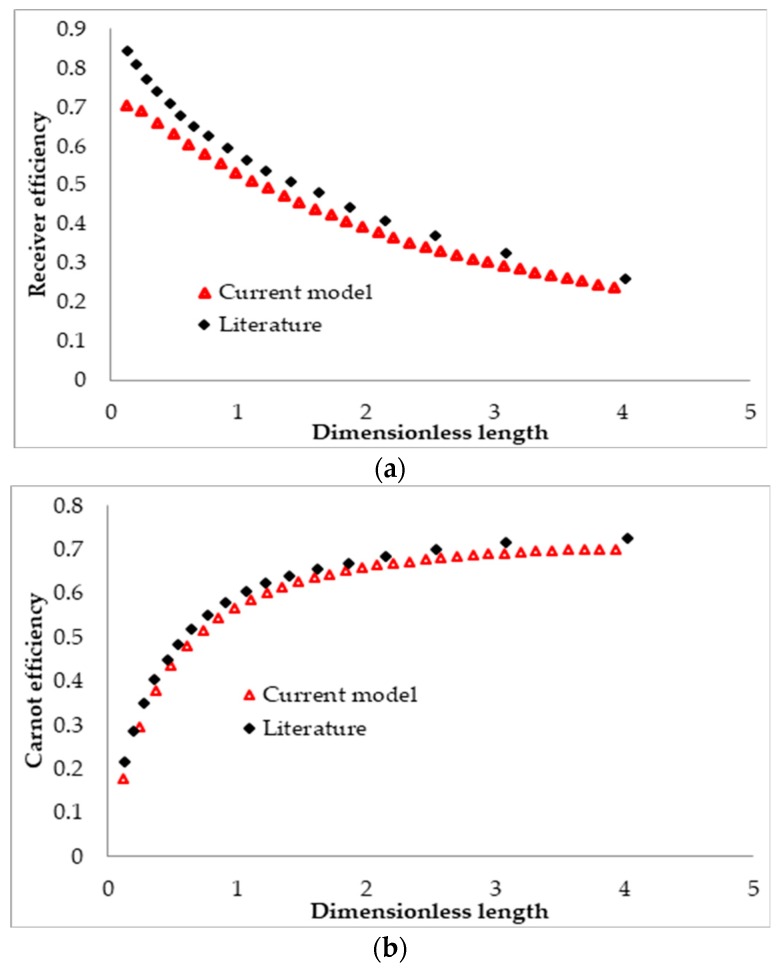

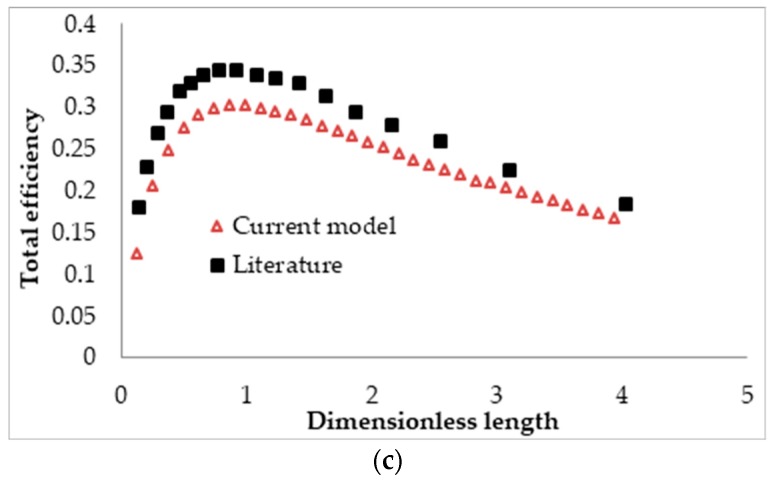
Comparison between the calculated efficiency using the current model and A. Veeraragavan’s model [51]: (**a**) receiver efficiency, (**b**) Carnot efficiency, and (**c**) total efficiency.

Trend of the receiver efficiency, the Carnot efficiency, and the total efficiency of a DAC receiver with its length was investigated and compared with those found by Veeraragavan et al. [51], as presented in Figure 4. A parametric sweep of the length ranging between 0.01 and 0.32 m with a step of 0.01 m was conducted that equates to a dimensionless length ranging between 0.123 and 3, similar to those in the literature. Equation (35) was used to define the dimensionless length of the receiver, whereas Equation (36) was used to calculate the efficiency.

(35) $$\bar{L}=\frac{L_{rec}}{Pe\cdot y_{rec}}$$

where Pe= Peclet number

As the figure shows, the investigated trend is almost similar to that of the literature. However, a slight under-prediction in the current study can be seen, possibly because of the governing equations used in this study in contrast to an analytical model using simple governing equations and solving combining homogeneous and particular integral solutions in the literature. Another reason may be the values used for the specific heat, density, and viscosity of Therminol in the current study. The values were extracted from [62], and are not available directly in the working literature.

(36) $$\eta_{tot}=\eta_{rec\;}\eta_{carnot}=\eta_{rec\;}\left(1-\frac{T_{amb}}{T_{out}}\right).$$

## 3. Results and Discussion

Due to the acceptable level of agreement between results of the current study and Veeraragavan’s model [51], this model can be considered validated. After validation, the model was further improved and several more factors were added. In order to increase the accuracy of the absorptive coefficient of the nanofluid, the model considers the refractive and absorptive indexes of both the base fluid and nanoparticles as wavelength-dependent. It also considers initial and inlet temperatures that are significantly higher than that of the ambient temperature. Due to the considerations of these high temperatures, the heat transfer equation is altered to include re-radiation of the nanofluid. The radiative heat loss is defined using Stefan–Boltzmann’s law and the convective heat loss is dependent on the Nusselt number and by default the inlet velocity and base fluid properties. In addition to the convective heat transfer coefficient being dependent on the Nusselt number, this model also considers the thermal resistance of a cover plate, taking into account its thermal conductivity and thickness. Additionally, the nanofluid absorption coefficient is a combination of the base fluid absorption coefficient and the nanoparticle coefficient. 

### 3.1. Effect of Receiver Length on the Efficiencies

**Figure 5:**
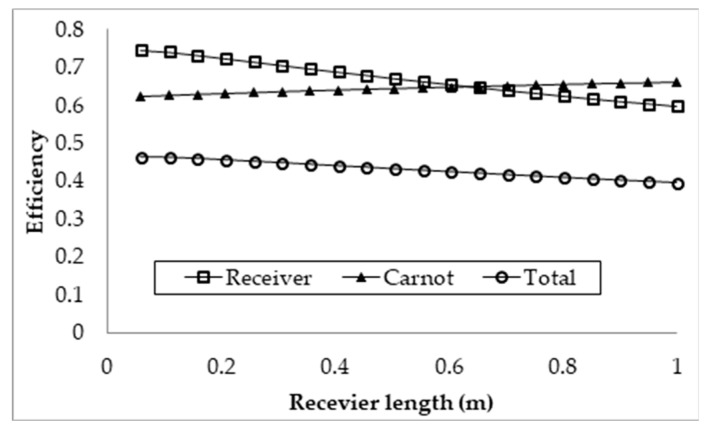
Effect of receiver length on the efficiency.

To run a parametric sweep of the receiver length, several parameters had to be arbitrarily set, those being a volume fraction of 1 × 10^−5^, an inlet velocity of 0.0021 m/s, and a solar concentration of 100×. The inlet velocity was set such that at the maximum receiver length of 1 m the peak temperature of the nanofluid did not exceed 1071 K. A high solar concentration was initially chosen to ensure that the temperature of the heat transfer fluid did not drop below its melting point. This can be an issue with low concentrations (for example, a concentration of 10×) because the nanofluid is initially at a temperature significantly higher than that of the ambient temperature, and the concentrated radiation is not great enough to maintain that temperature near the inlet of the receiver. To determine the overall performance, receiver efficiency, Carnot efficiency, and total efficiency were considered. The sweep was conducted over a receiver length range from 0.01 to 1 m with a step of 0.0495 m, as shown in Figure 5.

**Figure 6:**
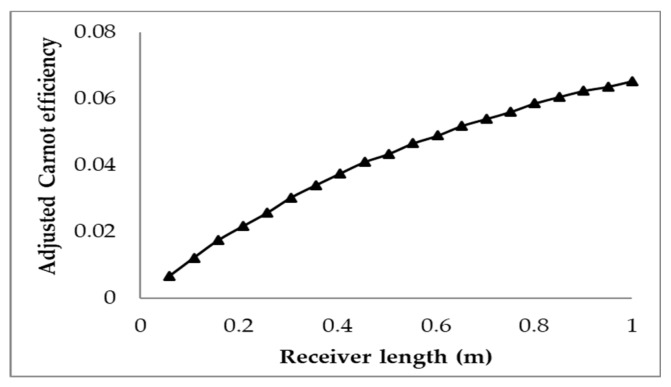
Adjusted Carnot efficiency for varying receiver length.

It can be seen from the plot that an increase in the receiver’s length results in a decrease in the receiver and total efficiencies, and an increase in the Carnot efficiency. The decrease in the receiver efficiency is due to an increasing surface area of the receiver and increasing fluid temperature with increasing length. This results in higher losses, as the losses are dominated by surface to ambient radiation, which is dependent on the surface area and the difference in receiver and ambient temperature to the power of four. Even though the rise in temperature results in an increase in the Carnot efficiency, it is not significant enough to counteract the decrease in receiver efficiency, resulting in a drop in the total efficiency. However, the Carnot efficiency does not pay enough attention to the rise in temperature achieved, which then implies that the most efficient receiver design is such that a negligible temperature rise occurs. This is not the case, as the entire objective of the receiver is to achieve a temperature rise. Hence, an adjusted Carnot efficiency (using the inlet temperature as the low temperature instead of the ambient temperature) is used to give a better indication of the trade-off between decreasing receiver efficiency and average temperature rise. This is illustrated in Figure 6.

From the plot, it can be seen that the adjusted efficiency continually increases with the receiver length. This reflects that the best trade-off between receiver efficiency and temperature rise occurs at the maximum receiver length. This implies that the longer the receiver is, the better the trade-off is (to a certain extent), which contradicts what the normal total efficiency implies. The trend of the plot is seen to be curved and not linear, which implies that, at a certain receiver length, it will reach a maximum and the efficiency will then decrease with increasing length. This point indicates where the trade-off between the receiver efficiency and the rise in temperature is at a maximum; if the length is further increased, the rise in average temperature will not be enough to offset the decrease in receiver efficiency.

### 3.2. Effect of Inlet Velocity on Collector Efficiency

**Figure 7:**
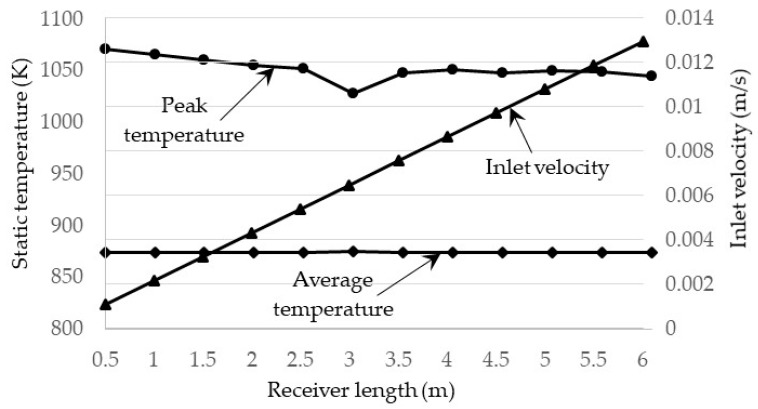
Inlet velocities and peak temperatures for different receiver lengths at a constant exposure time.

Another factor that affects the performance of the receiver is the inlet velocity of the heat transfer fluid. An increase in velocity allows for longer receiver lengths but also increases the Nusselt number and by extension the convective heat transfer coefficient, resulting in higher losses. However, as the heat transfer fluid in question is of such a high temperature, the thermal losses are dominated by the radiative losses; as such, the increase in the convective heat transfer coefficient results in only a small drop in efficiency. Given that Li_2_CO_3_–K_2_CO_3_ is at an even higher temperature, it is expected that the inlet velocity will have an even smaller effect on the total efficiency than it did with the solar salt. To investigate this, the velocity and receiver length were both varied to achieve a constant exposure time, chosen as 463 s. This time was selected from determining at what velocity for a receiver length of 0.5 m the peak temperature of the nanofluid would be equal to its upper temperature limit if 1071 K. The exposure time is given simply as the receiver length divided by the inlet velocity. This exposure time was then held constant for receiver lengths ranging from 0.5 to 6 m with a step of 0.5 m. The receiver lengths and their associated inlet velocities are summarized in Figure 7. It should be noted that this simulation was conducted for a receiver height of 0.0908 m, a volume fraction of 1 × 10^−5^, and a solar concentration of 100×.

**Figure 8:**
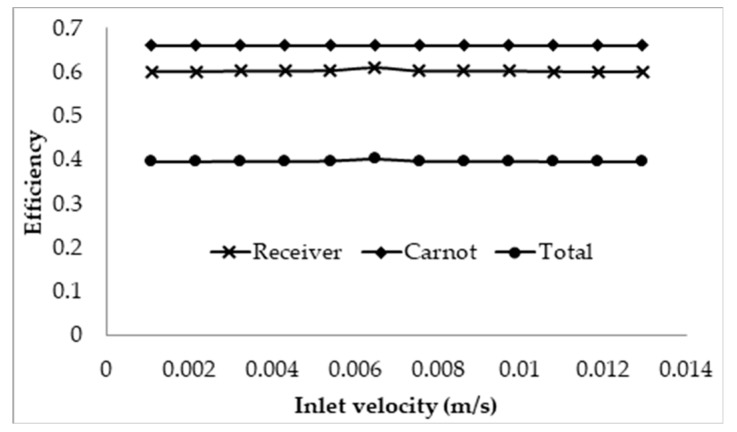
Effect of inlet velocity on the efficiency of the receiver at a fixed peak temperature.

The receiver efficiency, Carnot efficiency, and total efficiency are illustrated in Figure 8. It can be seen that each type of efficiency is constant and unchanging with the change in velocity. This shows that, at very high receiver temperatures, the change in velocity has a negligible effect on the overall performance of the receiver as the thermal losses are dominated by the surface to ambient radiation losses. The same can be said for the adjusted total efficiency that there is negligible change with inlet velocity. This can be concluded since, in Figure 7, it can be seen that the average outlet temperature does not change, which in turn means that the adjusted Carnot efficiency does not change, resulting in no change in the total adjusted efficiency.

**Figure 9:**
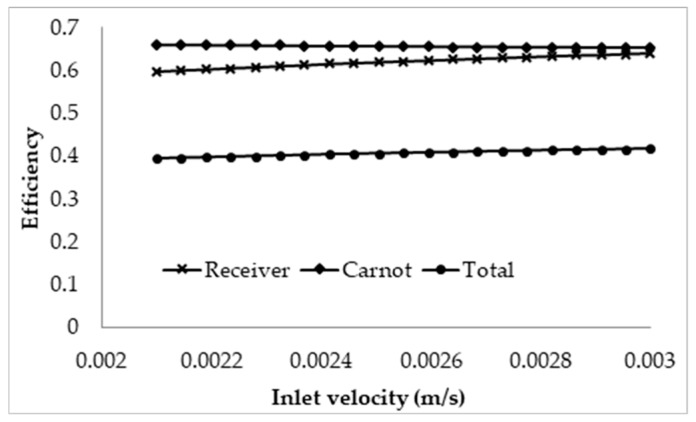
Effect of inlet velocity on the efficiency of the receiver of fixed length of 1 m.

The effects of the inlet velocity were also investigated by keeping the receiver length constant and then increasing the velocity. The receiver was set with the same parameters and the length was arbitrarily chosen as 1 m. For this length, the lowest velocity will be such that the peak temperature of the nanofluid is approximately the upper temperature limit of 1071 K. This corresponds to a velocity of 0.0021 m/s; the velocity is then ranged from this value to 0.003 m/s with a step of 4.5 × 10^−5^ m/s. The results are illustrated in Figure 9.

**Figure 10:**
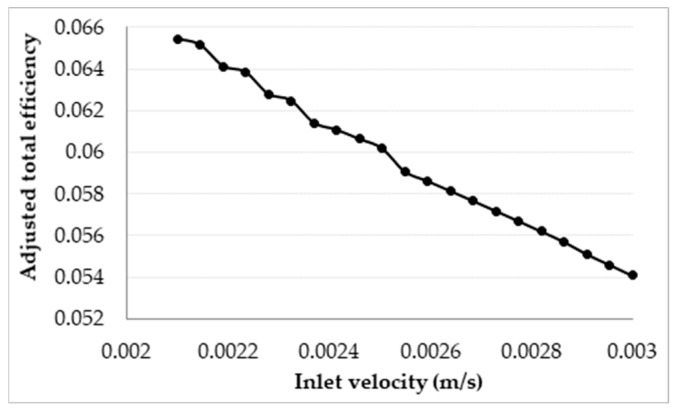
Adjusted total efficiency for a varying inlet velocity at a fixed receiver length of 1 m.

The results depicted are somewhat controversial, as it shows that, by increasing the inlet velocity, the overall efficiency of the receiver is actually increased. This effect can again be attributed to the dominating nature of the surface to ambient radiation losses; with an increase in the velocity, the exposure time is reduced, resulting in a decrease in the temperature rise across the receiver. Even though an increase in the velocity increases the convective losses of the receiver, the drop in the temperature rise results in a much larger drop in the overall thermal losses, causing the receiver to become more efficient. However, when considering the adjusted efficiency as depicted in Figure 10, it can be seen that the total efficiency decreases with an increase in the velocity, with the most efficient design being such that the peak temperature is approximately equal to the upper temperature limit of the nanofluid.

Therefore, while for a fixed receiver length an increase in velocity results in a decrease in the adjusted total efficiency of the receiver, it has a negligible effect on the receiver if the length and velocity are adjusted such that the peak temperature of the nanofluid is the same as the upper temperature limit.

### 3.3. Effect of Volume Fractions, f_v_, on the Efficiencies

**Figure 11:**
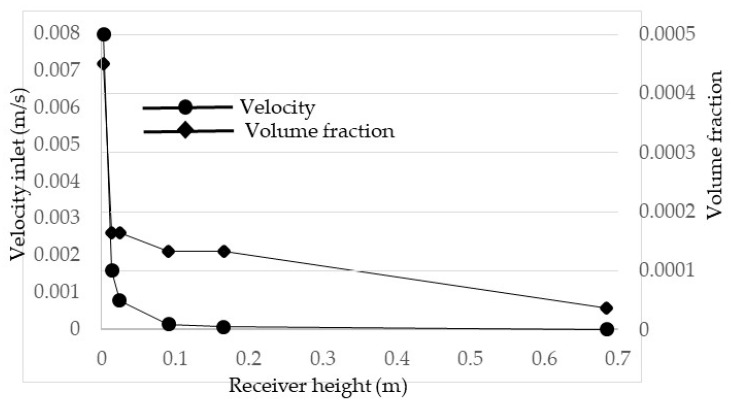
Relevant velocities for volume fractions for a constant peak temperature.

The volume fraction of the nanoparticles suspended in the base fluid is another important factor that has to be considered because it directly influences the absorptivity of the nanofluid. It was seen that an increase in the volume fraction of nanoparticles significantly increased the absorption coefficient of the nanofluid, resulting in a shallower receiver required to absorb 99% of the radiation [6]. An interesting trend was also observed where the total efficiency of the receiver with solar salt decreased with an increasing volume fraction, but only up to a point when it subsequently started to increase. The adjusted total efficiency also showed a different trend where a linear increase in efficiency was evident with increasing volume fraction, implying that the more efficient design was one with a high volume fraction. The aim of this section is to investigate whether these same trends are apparent for a different base fluid with a different thermal conductivity and specific heat, and a higher operating temperature. To accomplish this, parametric sweeps of the receiver length, the same as that conducted in the previous receiver length section, were conducted for different volume fractions and receiver heights. The solar concentration was kept at 100× and the inlet velocities were adjusted such that, at the maximum length of 1 m, the peak temperature of the receiver was equal to the upper temperature limit of the base fluid. This is summarized in Figure 11.

**Figure 12:**
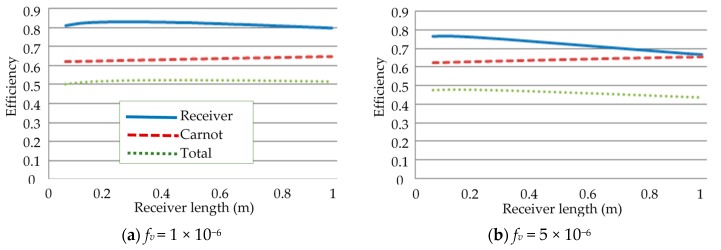

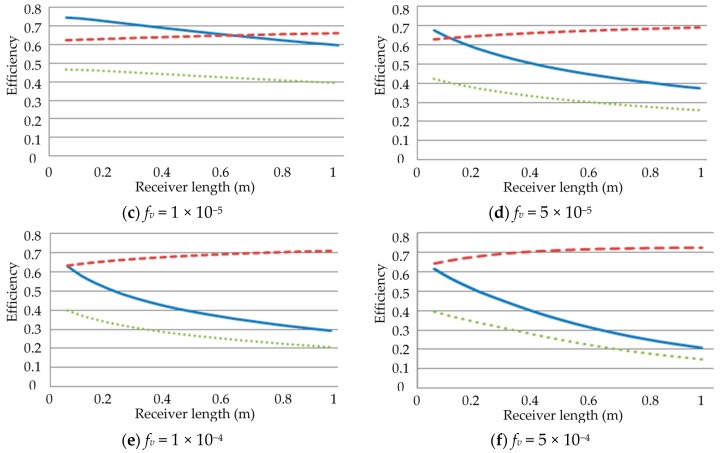
Effect of volume fraction (*f_v_*) on the efficiencies.

Figure 12 illustrates the receiver, Carnot, and total efficiencies of different volume fractions of nanoparticles over a range of receiver lengths. It can be seen that the most efficient receivers are those with low volume fractions with total efficiencies as high as 50%. In addition, the total efficiency barely changes with increasing receiver length for the lowest volume fraction but then tends to decrease with increasing length, more and more significantly as the volume fraction increases. This is due to the attenuation of the solar radiation through the nanofluid; at low volume fractions, the radiation is attenuated less, and the temperature of the nanofluid therefore does not rise as much, as the energy is absorbed over a larger volume of fluid, resulting in lower thermal losses and higher receiver efficiencies. The opposite is true for high volume fractions; the solar radiation is absorbed over a smaller volume for fluid and therefore results in higher temperatures and higher thermal losses. The drop in total efficiency is due to the drop in receiver efficiency being greater than that of the increase in Carnot efficiency. To better understand the balance between receiver efficiency and temperature rise, the adjusted efficiency is also considered.

**Figure 13:**
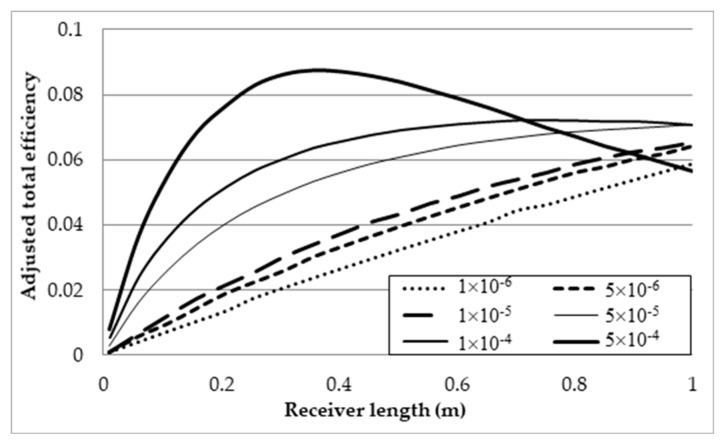
Adjusted total efficiency at different volume fractions.

Figure 13 depicts the adjusted total efficiency for each volume fraction over the receiver length range. From the plot, it can be seen that the maximum efficiency occurs at the maximum length of 1 m for volume fractions up to 5 × 10^−5^. After this point, an optimal receiver length becomes apparent and decreases with increasing volume fraction. This point indicates the receiver length that will provide the best trade-off between reduced receiver efficiency and temperature rise, after which point the drop in receiver efficiency becomes too great to justify the rise in temperature.

**Figure 14:**
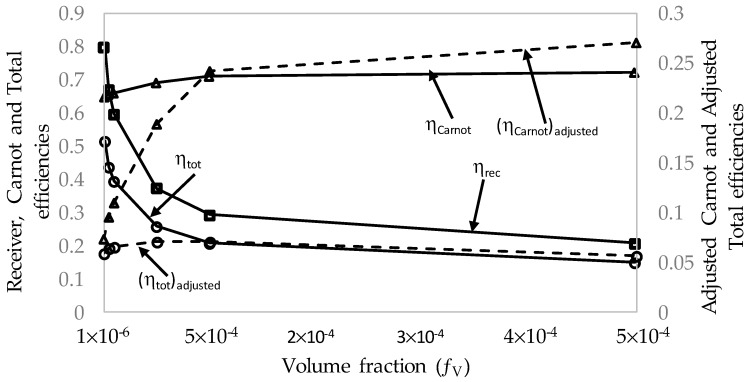
Efficiencies (*η*) for different volume fractions, *f_v_*, at a fixed peak temperature, *T_peak_* = 1071 K, and maximum receiver length, *L*_max_ = 1 m.

To directly compare the effects that the volume fraction has on the overall performance of the receiver, the results of each volume fraction at the maximum receiver length, 1 m, and peak temperature, 1071 K, are compared and presented in Table 3 (refer the first six rows) and Figure 14.

**Figure 15:**
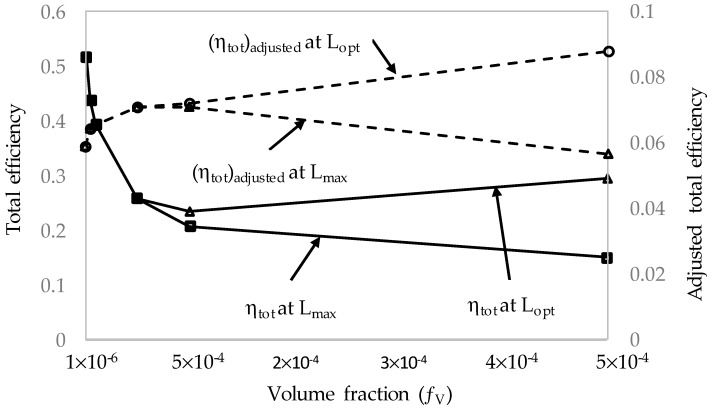
Comparison of total efficiency and total adjusted efficiency at a fixed peak temperature, *T_peak_*, for different volume fractions, *f_v_*, at maximum receiver length, *L*_max_, and optimum receiver length, *L_opt_*.

Figure 14 shows that the receiver efficiency and total efficiency exponentially decrease with increasing volume fraction, and the adjusted total efficiency peaks around a volume fraction of 5 × 10^−5^ to 1 × 10^−4^ instead of showing a linear relationship. For volume fractions of 1 × 10^−4^ and 5 × 10^−4^, the optimal length is not equal to the maximum length. Therefore, instead of using the maximum receiver length as the point of reference, the peak adjusted total efficiency should instead be the point of reference. These values are depicted in the same table, Table 3 (refer the first four rows + the last two rows), and Figure 15.

By considering the peak adjusted efficiency as the point of reference, it can be seen that the volume fractions 1 × 10^−4^ and 5 × 10^−4^ are significantly more efficient. The total efficiency declines sharply initially with increasing volume fraction, levels off, and then begins to increase. The adjusted efficiency also has an almost linear relationship with the volume fraction, with a steep increase initially.

### 3.4. Effect Solar Concentration

**Figure 16:**
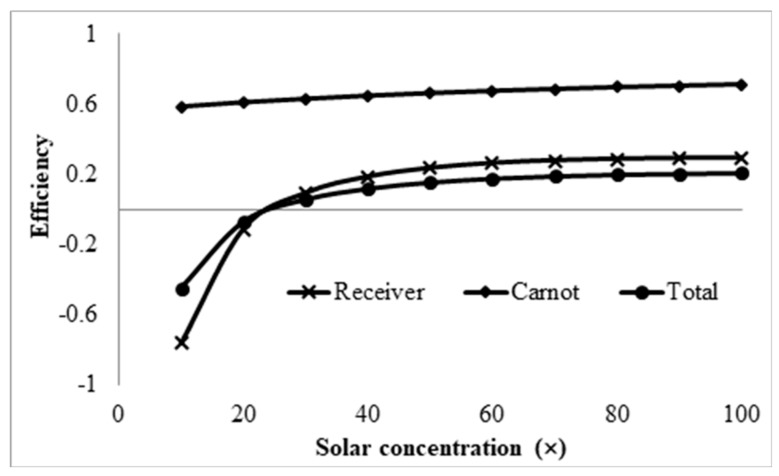
Effect of solar concentrations on receiver, Carnot, and total efficiencies.

The final parameter investigated is the solar concentration. To do this, the receiver height, volume fraction, and receiver length were set at 0.0142 m, 0.0001, and 1 m, respectively. The inlet velocity was set such that, at the maximum length of 1 m, the peak temperature of the nanofluid was equal to the upper temperature limit of the base fluid. This corresponded to an inlet velocity of 0.0248 m/s. The overall performance of the receiver is illustrated in Figure 16.

**Figure 17:**
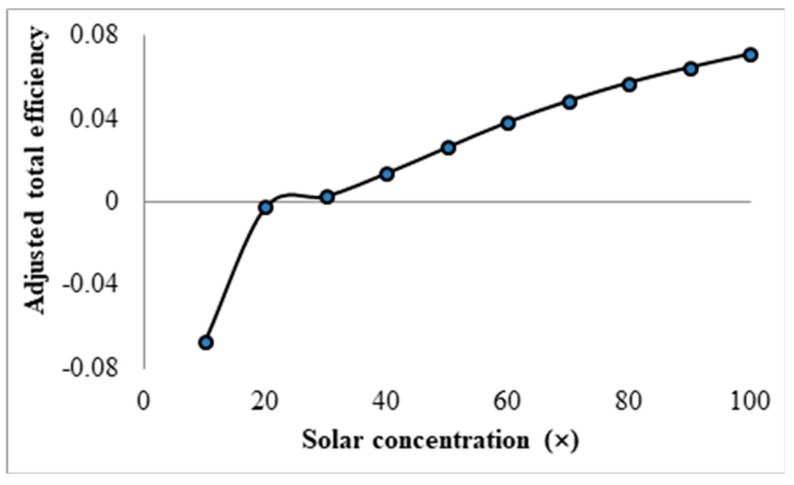
Effect of solar concentrations on adjusted total efficiency.

From the plot, it can be seen that, at low solar concentrations (<25×), the receiver efficiency is negative because the receiver is losing more energy than it is gaining from the concentrated solar radiation. This is due to the significantly high initial temperature of the nanofluid, as high solar concentrations are required to just maintain this temperature. Furthermore, from the plot, it can be seen that the receiver and Carnot efficiencies both increase with the solar concentration, implying that increasing the solar concentration does not have an adverse effect on the receiver. The adjusted total efficiency is also considered to verify whether this is the case. From Figure 17, it can be seen that the adjusted total efficiency does increase almost linearly with solar concentration. This further implies that the higher the solar concentration is, the more efficient the receiver is.

**Figure 18:**
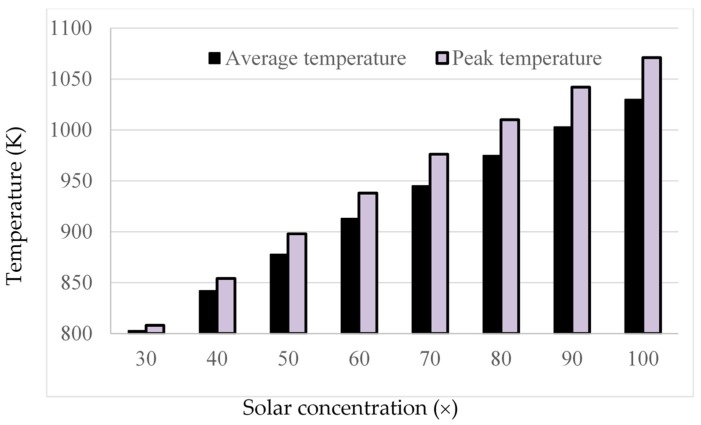
Average and peak temperatures for different solar concentrations.

However, when comparing the average and peak temperatures of the receiver at different solar concentrations, an interesting trend becomes apparent. As shown in Figure 18, the difference between the peak temperature and the average outlet temperature actually increases with increasing solar concentration. 

**Figure 19:**
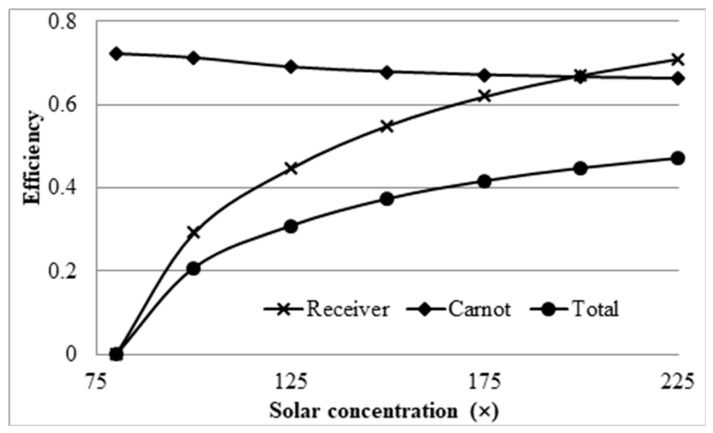
Effect of solar concentration on efficiency at a constant peak temperature.

This trend therefore implies that, if the peak temperature were kept constant, the Carnot efficiency would actually decrease with increasing solar concentration, which in turn implies that there would be an optimal solar concentration that provides the best trade-off between Carnot efficiency and receiver efficiency. This was investigated using the same parameters as already stated and varying the inlet velocity to obtain a constant peak temperature for a range of solar concentrations. The results are summarized in Figure 19.

The plot does in fact reflect what is expected, as it can be seen that the Carnot efficiency decreases. However, the rise in receiver efficiency is great enough to overcome this decrease and result in a net increase in the total efficiency with increasing solar concentration. It should be noted that the solar concentration starts at 80×, since, for any concentrations less than this value, the peak temperature of 1071 K could not be achieved. This plot does not give an accurate representation of the overall performance of the receiver because it does not place enough emphasis on the temperature rise of the receiver. When considering the adjusted Carnot efficiency, a different trend is observed.

**Figure 20:**
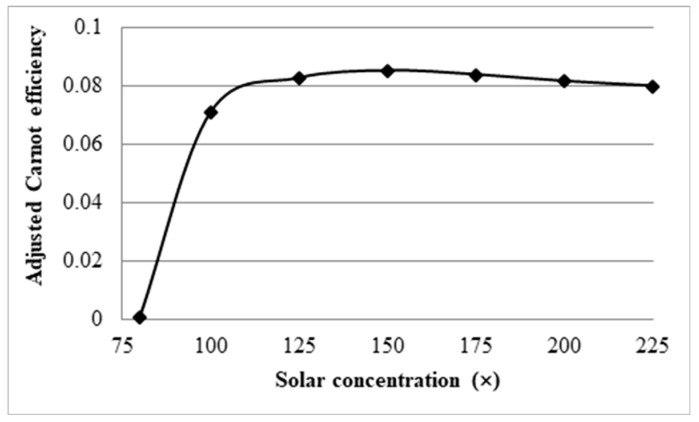
Adjusted total efficiency at a constant peak temperature.

From Figure 20, it can be seen that there is a peak in the adjusted total efficiency at a solar concentration of approximately 150×. After this point, the drop in the Carnot efficiency is not justified by the increase in the receiver efficiency. As such for a receiver with a volume fraction of 1 × 10^−4^ and a length of 1 m, the optimal solar concentration is approximately 150×.

### 3.5. Comparison with Solar Salt Nanofluid

**Figure 21:**
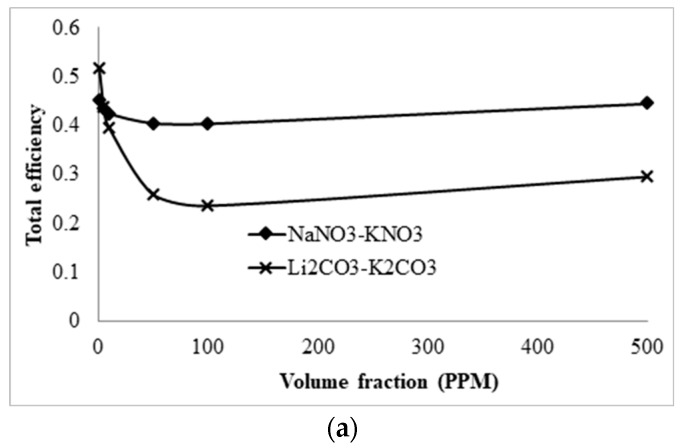

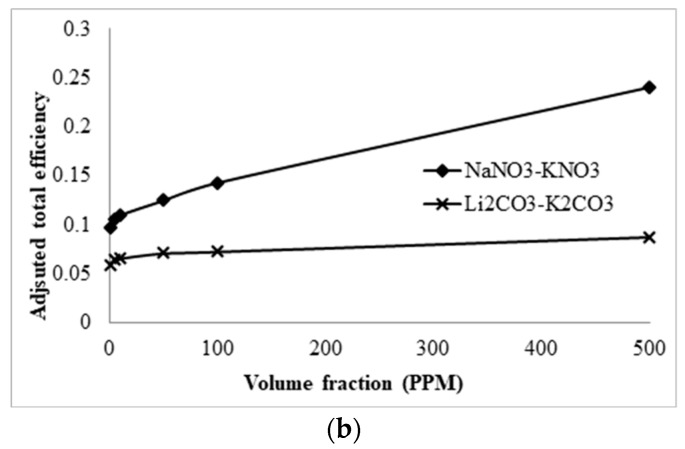
Comparison of the (**a**) total efficiency and (**b**) adjusted total efficiency between NaNO_3_–KNO_3_ and LiCO_3_–K_2_CO_3_ molten salt nanofluids.

The effect of Li_2_CO_3_–K_2_CO_3_ molten salt nanofluid on the total efficiency and the adjusted total efficiency of the receiver in comparison to that of solar salts was investigated, as presented in Figure 21.

The plots show that both molten salts, even though solar salt is more efficient, follow similar trends. The difference in efficiencies is expected as LiCO_3_–K_2_CO_3_ operates at higher temperature and is therefore subject to higher thermal losses. The reason, therefore, for choosing LiCO_3_–K_2_CO_3_ over solar salt is that the higher the average temperature it can achieve, the more desirable for thermal storage it will be, as a greater amount of energy can be stored for a smaller volume of liquid.

## 4. Conclusions

A molten salt nanofluid, with the composition of graphite as the nanoparticles in LiCO_3_–K_2_CO_3_ base fluid, was studied computationally for the receiver of a DAC using a validated CFD model. Several parameters including the receiver length, inlet velocity, volume fraction, and the concentration of the incident solar radiation were investigated for the nanofluid to explore their effects on the overall energy performance of the receiver. The results are discussed extensively in this paper. The following conclusions can be drawn from the study:

⮚ Investigation into the receiver length and nanofluid volume concentration shows that, at high operating temperature, the receiver efficiency (from ≈0.75 to 0.6) and total efficiency (from ≈0.48 to 0.4) are decreasing, while the Carnot efficiency (from ≈0.615 to 0.68) is increasing slightly with receiver length. An adjusted Carnot efficiency (inlet temperature as the low temperature instead of the ambient temperature) shows that the rise in average temperature of the HTF is exponential with receiver length to a certain maximum receiver length, beyond which the temperature rise is not enough to offset the receiver efficiency decrease.
⮚ Using the normal Carnot efficiency, it was shown that an increase in heat transfer fluid (HTF) velocity resulted in no apparent effect on the overall efficiency ($\eta_{Carnot}$ = 0.66, $\eta_{rec}=0.6,\;\mathrm{and}\;\eta_{tot}=0.4)$ at a fixed peak temperature (1071 K) receiver, and resulted in a slight increase in the overall efficiency of a fixed length (1 m) receiver as outlet temperature dropped. However, the adjusted total efficiency of the fixed length receiver was found to be decreased with the increase of inlet velocity. This implied that the most efficient receiver was one that had very low HTF velocity, as that resulted in a higher temperature, which is true to a certain extent, until the upper temperature limit of the nanofluid was reached.
⮚ A range of volume fraction (from 1 × 10^−6^ to 5 × 10^−4^) of nanoparticles was investigated over a range of different receiver lengths with initial results indicating that the most efficient receiver ($\eta_{tot}\approx \;0.5$) was that with the lowest volume fraction and at the shortest receiver length. That again was not an accurate representation as when considering the adjusted total efficiency. It was found that the efficiency of the receiver increased with volume fraction and receiver length to an extent, as an optimal receiver length became evident. It was discovered that the higher the volume fraction was, the shorter the optimal length became. A higher volume fraction resulted in a higher average outlet temperature and greater efficiency, but a greater susceptibility to heat loss to the ambient.
⮚ Initial investigations into the effects of solar concentration revealed that both the normal Carnot efficiency (from 0.6 to 0.7) and the total efficiency (from −0.55 to 0.2), and the adjusted Carnot efficiency and the adjusted total efficiency, are gradually increased with an increase in the solar concentration (from 10× to 100×). An interesting trend was observed, however, where the difference between the average and peak temperatures of the receiver actually increased by 5–41 K by increasing solar concentration from 30× to 100×. This discovery implies that, if the peak temperature were kept constant by balancing the receiver length and inlet velocity, the average temperature would actually decrease at some point with increasing solar concentration, resulting in a decrease in the Carnot efficiency and a drop in the overall efficiency of the receiver, which contradicts the initial results. By keeping the peak temperature constant, an optimal solar concentration was indeed discovered when considering the adjusted Carnot efficiency. For a receiver with a volume fraction of 1 × 10^−4^ and a length of 1 m, the optimal solar concentration is approximately 150×.
⮚ A comparative study shows that solar salt is superior to the lithium carbonate salt because of the higher total efficiency of the collector. However, LiCO_3_–K_2_CO_3_ will be more desirable for thermal storage, as a greater amount of energy can be stored for a smaller volume of liquid at higher operating temperature.

## Figures and Tables

**Table 1:** Parameters used in the computational model.

Parameters	Values
Height of receiver, $y_{rec}$	0.0142 (m)
Width of receiver, $w_{rec}$	0.005 (m)
Length of receiver, $l_{rec}$	1.0 (m)
$\mathrm{Thermal}\;\mathrm{conductivity}\;\mathrm{of}\;\mathrm{base}\;\mathrm{fluid},\;k_{bf}$	0.135 (W/mK)
$\mathrm{Specific}\;\mathrm{heat}\;\mathrm{of}\;\mathrm{base}\;\mathrm{fluid},\;C_{p,bf}$	1600 (J/kgK)
$\mathrm{Density}\;\mathrm{of}\;\mathrm{base}\;\mathrm{fluid},\;\rho_{bf}$	2100 (kg/m^3^)
$\mathrm{Density}\;\mathrm{of}\;\mathrm{nanoparticles},\;\rho_{p}$	2100 (kg/m^3^)
$\mathrm{Dynamic}\;\mathrm{viscosity}\;\mathrm{of}\;\mathrm{base}\;\mathrm{fluid},\;\mu_{bf}$	0.00328 (Pa.s)
$\mathrm{Inlet}\;\mathrm{temperature},\;T_{in}$	780 (K)
$\mathrm{Inlet}\;\mathrm{velocity},\;v_{in}$	0.0034 (m/s)
Speed of light, c	299,792,458 (m/s)
Diameter of nanoparticles, D	50 × 10^−9^ (m)
$\mathrm{Volume}\;\mathrm{fraction}\;\mathrm{of}\;\mathrm{nanoparticles},\;V_{f,p}$	0.00005
$\mathrm{Attenuation}\;\mathrm{constant},\;S_{att}$	0.73
Solar concentration (times), Conc	100×
$\mathrm{Solid}\;\mathrm{angle}\;\mathrm{of}\;\mathrm{the}\;\mathrm{sun}\;\mathrm{from}\;\mathrm{Earth},\;\Omega_{s}$	6.8 × 10^−5^ (steradians)
Planck’s constant, h	6.62606957 × 10^−34^ (J.s)
$\mathrm{Boltzmann}\;\mathrm{constant},\;k_{b}$	1.3806488 × 10^−23^ (J/K)
$\mathrm{Temperature}\;\mathrm{of}\;\mathrm{the}\;\mathrm{sun},\;T_{sun}$	5780 (K)
$\mathrm{Ambient}\;\mathrm{temperature},\;T_{amb}$	297K
$\mathrm{Refractive}\;\mathrm{index}\;\mathrm{of}\;\mathrm{nanoparticle},\;n_{p}$	2.72
$\mathrm{Thickness}\;\mathrm{of}\;\mathrm{top}\;\mathrm{plate},\;t_{fq}$	0.01 (m)
$\mathrm{Thermal}\;\mathrm{conductivity}\;\mathrm{of}\;\mathrm{top}\;\mathrm{plate},\;k_{fq}$	1.3 (W/m.K)
$\mathrm{Absorptive}\;\mathrm{index}\;\mathrm{of}\;\mathrm{nanoparticle},\;k_{abs,p}$	1.31
$\mathrm{Refractive}\;\mathrm{index}\;\mathrm{of}\;\mathrm{base}\;\mathrm{fluid},\;n_{bf}$	1.63
$\mathrm{Absorptive}\;\mathrm{index}\;\mathrm{of}\;\mathrm{base}\;\mathrm{fluid},\;k_{abs,bf}$	3.86 × 10^−8^
$\mathrm{Lower}\;\mathrm{limit}\;\mathrm{of}\;\mathrm{wavelength}\;\mathrm{range},\;\lambda_{\min}$	200 × 10^−9^ (m)
$\mathrm{Upper}\;\mathrm{limit}\;\mathrm{of}\;\mathrm{wavelength}\;\mathrm{range},\;\lambda_{\max}$	2500 × 10^−9^ (m)
$\mathrm{Combined}\;\mathrm{radiative}\;\mathrm{and}\;\mathrm{convective}\;\mathrm{heat}\;\mathrm{loss}\;\mathrm{coefficient},\;h_{total}$	13.5 (W/m^2^K)

**Table 2:** Variables used in the computational model.

Variables	Expression
Concentrated normally incident solar radiation distribution, $I_{0}$	$S_{att}c\Omega_{s}\frac{2h_{p}c^{2}}{\lambda^{5}}\frac{1}{e^{\frac{hc}{\lambda k_{B}T_{sun}}}-1}$
Concentrated normally incident solar radiation, $P_{y,0}$	$\int_{\lambda_{miin}}^{\lambda_{\max}}I_{0}d\lambda$
Distance from surface of receiver, $y_{1}$	$y_{rec}-z$
Relative complex refraction index of nanofluid, $m_{nf}$	$\frac{n_{p}+ik_{abs,p}}{n_{bf}+ik_{abs,bf}}$
Total mass of the nanofluid, M	$\frac{m_{nf}^{2}-1}{m_{nf}^{2}+2}$
Absorption efficiency, $Q_{abs}$	4πDλIm(M(1+(πDλ)2115MM2))
Scattering efficiency, $Q_{scant}$	$\frac{8}{3}{\left(\frac{\pi D}{\lambda }\right)}^{4}{\left|M\right|}^{2}$
Absorption coefficient of nanoparticles, $\kappa_{abs}$	$\frac{3V_{f,p}\left(Q_{abs}+Q_{scat}\right)}{2D}$
Absorption coefficient of base fluid, $\kappa_{abs,bf}$	$\frac{4\pi \kappa_{abs}}{\lambda }$
Spectral flux, $I_{y}$	$I_{0}e^{-\kappa_{abs,total}y_{1}}$
Divergence of the spectral flux, $P_{y}$	$\int_{\lambda_{\min}}^{\lambda_{\max}}I_{y}d\lambda$
Volumetric heat release, $Q_{source}$	$-\frac{dP_{y}}{dy_{1}}$
Average outlet temperature, $T_{ave,out}$	ave,out(T)
Efficiency of receiver, $\eta_{rec}$	$\frac{\rho_{bf}v_{in}y_{rec}w_{rec}C_{p,bf}\left(T_{out}-T_{in}\right)}{P_{y,0}l_{rec}w_{rec}}$

**Table 3:** Receiver results of each volume fraction at the maximum receiver length (first six rows) and maximum adjusted total efficiency (first four rows + last two rows).

*f_v_*	*L_rec_* (m)	*v_in_* (m/s)	*T_avg_* (K)	*η_rec_* (%)	*η_Carnot_* (%)	(*η_Carnot_*)*_adjusted_* (%)	*T_peak_* (K)	*η_Total_* (%)	(*η_Total_*)*_adjusted_* (%)
1 × 10^−6^	1	0.000575	842	0.798	0.647	0.074	1071	0.517	0.059
5 × 10^−6^		0.0021	863	0.668	0.656	0.096		0.438	0.064
1 × 10^−5^		0.0021	876	0.597	0.661	0.110		0.395	0.065
5 × 10^−5^		0.0026	962	0.374	0.691	0.189		0.259	0.071
1 × 10^−4^		0.0026	1029	0.293	0.711	0.242		0.208	0.071
5 × 10^−4^		0.0072	1069	0.209	0.722	0.270		0.151	0.057
1 × 10^−4^	0.7525	0.0026	994	0.336	0.701	0.215	1053	0.236	0.072
5 × 10^−4^	0.3565	0.0072	984	0.422	0.698	0.207	1001	0.295	0.0878

## Nomenclature

$A_{r}$	Collector area (m^2^)
*a*_o_ and *a*_1_	Constant parameters
C	Concentration factor
c	Speed of light (m/s)
$c_{p}$	Specific heat (W/mK)
D	Particle diameter (m)
$f_{v}$	Volume fraction of nanoparticles
h	Planck’s constant
$h_{nf}$	Nanofluid heat transfer coefficient (W/mK)
$h_{total}$	Overall heat transfer coefficient (W/mK)
$I_{0}$	Incident radiation (W/m^2^)
$I_{s}$	Scattered irradiation (W/m^2^)
k	Thermal conductivity (W/m^2^K)
$k_{b}$	Boltzmann constant
$k_{nf}$	Thermal conductivity of nanofluid (W/m^2^K)
$k_{tp}$	Thermal conductivity of top plate (W/m^2^K)
L	Characteristic length taken as receiver height (m)
$L_{rec}$	Length of receiver (m)
m	Relative complex refractive index of the nanofluid =SnpSbf
$\dot{m}$	Mass flow rate (kg/s)
N	Number of scattering particles in the beam path
$n_{np}$	Refractive index
Nu	Nusselt number
*P_e_*	Peclet number
$P_{0}$	Solar irradiance integrated over solar wavelength range (W/m^2^)
Pr	Prandtl number
Q	Heat source (W)
Re	Reynolds number
$S_{att}$	Attenuation constant, which accounts for the average attenuation through Earth’s atmosphere
$S_{bf}$	Complex refractive index of the base fluid
$S_{j}$	Strength of the j th resonant vibration mode
$S_{np}$	Complex refractive index of the nanoparticles
T	Temperature (K)
t	Thickness of top plate (m)
$T_{amb}$	Ambient temperature (K)
$T_{in}$	Inlet temperature (K)
$T_{out}$	Average outlet temperature (K)
$T_{sun}$	Temperature of the sun (K)
u	Fully developed velocity (m/s)
$v_{in}$	Inlet nanofluid velocity (m/s)

## Greek Symbols

$a_{np}$	Absorptive index
ϑ	Scattering angle
$\Omega_{s}$	Solid angle of the sun as seen from Earth (steradian)
$\delta_{j}$	Damping parameter of the j th resonant vibration mode
$\phi_{\max}$	Maximum packing concentration
$\eta_{Carnot}$	Carnot efficiency (%)
$\eta_{rec}$	Receiver efficiency (%)
$\eta_{tot}$	Total efficiency (%)
λ	Radiation wavelength (m)
$\lambda_{j}$	Characteristic wavelength of the j th resonant vibration mode (m)
θ	Incident angle (rad)
ρ	Density (kg/m^3^)
